# Analysis of Bulk RNA Sequencing Data Reveals Novel Transcription Factors Associated With Immune Infiltration Among Multiple Cancers

**DOI:** 10.3389/fimmu.2021.644350

**Published:** 2021-08-20

**Authors:** Lei Liu, Qiuchen Zhao, Chao Cheng, Jingwen Yi, Hongyan Sun, Qi Wang, Weili Quan, Yaqiang Xue, Luguo Sun, Xianling Cong, Yi Zhang

**Affiliations:** ^1^National Engineering Laboratory for Druggable Gene and Protein Screening, Northeast Normal University, Changchun, China; ^2^Center of Genome Analysis, ABLife BioBigData Institute, Wuhan, China; ^3^College of Life Sciences, Wuhan University, Wuhan, China; ^4^Research Center of Agriculture and Medicine Gene Engineering of Ministry of Education, Northeast Normal University, Changchun, China; ^5^Tissue Bank, China–Japan Union Hospital, Jilin University, Changchun, China

**Keywords:** immune infiltration, pan-cancers, transcription factor, immune cell types, FOXM1, MYBL2, TAL1, ERG

## Abstract

Tumor-infiltrating immune cells shape the tumor microenvironment and are closely related to clinical outcomes. Several transcription factors (TFs) have also been reported to regulate the antitumor activity and immune cell infiltration. This study aimed to quantify the populations of different immune cells infiltrated in tumor samples based on the bulk RNA sequencing data obtained from 50 cancer patients using the CIBERSORT and the EPIC algorithm. Weighted gene coexpression network analysis (WGCNA) identified eigengene modules strongly associated with tumorigenesis and the activation of CD4+ memory T cells, dendritic cells, and macrophages. TF genes *FOXM1*, *MYBL2*, *TAL1*, and *ERG* are central in the subnetworks of the eigengene modules associated with immune-related genes. The analysis of The Cancer Genome Atlas (TCGA) cancer data confirmed these findings and further showed that the expression of these potential TF genes regulating immune infiltration, and the immune-related genes that they regulated, was associated with the survival of patients within multiple cancers. Exome-seq was performed on 24 paired samples that also had RNA-seq data. The expression quantitative trait loci (eQTL) analysis showed that mutations were significantly more frequent in the regions flanking the TF genes compared with those of non-TF genes, suggesting a driver role of these TF genes regulating immune infiltration. Taken together, this study presented a practical method for identifying genes that regulate immune infiltration. These genes could be potential biomarkers for cancer prognosis and possible therapeutic targets.

## Introduction

Cancer immunotherapy involves the use of drugs to either relieve the immune suppression of the tumor microenvironment or strengthen the immune system to eliminate cancer cells; it indeed provides enormous durable clinical benefits to patients with late-stage cancer across many tumor types (1). Tumor-infiltrating immune cells can shape the tumor microenvironment and are closely linked to immunotherapy. Compositions, localizations, and even orientations of tumor-infiltrating immune cells can influence the efficacy of anticancer immune responses (2, 3). The quantitation of the immune contexture is crucial to not only the prognosis (4) but also the checkpoint-blocker-based monotherapy or combination therapy (5). Many computation methods have been developed to study the immune cell composition dynamics during tumorigenesis, such as EPIC (6), TIMER (7), and CIBERSORT (8). Based on the gene expression profiles in the bulk tumor tissue and the specific gene expression profiles of various immune cells, these methods allow the quantification of immune cell composition by the conventional gene profiling methods, including bulk RNA-seq.

Several transcription factors (TFs) have been reported to suppress the antitumor immune response in various solid tumors. The expression pattern of forkhead box protein P3 (FOXP3) in tumor-infiltrating lymphocytes of primary cutaneous melanoma (PCM) suggests that FOXP3-expressing lymphocytes may suppress the local anti-PCM immune response in the microenvironment, thus favoring melanoma progression (9). A recent study in human breast cancer identified FOXP1 as an important negative regulator of antitumor immune responses *via* its control of the chemokine expression (10). Signal transducer and activator of transcription 3 (STAT3) is constitutively activated in both tumor and immune cells, representing a promising target for cancer therapy. STAT3 directly regulates the expression of oncogenes and triggers tumor progression and also induces tumor-induced immunosuppression that indirectly promotes human cancer growth (11). TF c-Maf controls the immunosuppressive macrophage polarization and function in cancer *via* the transcriptional regulation of M2-related genes, serving as a metabolic checkpoint, overexpressing in tumor-associated macrophages (TAMs), and regulating TAM immunosuppressive function. The knockout of c-Maf in myeloid cells contributes to decreased tumor burden with improved antitumor T-cell immunity (12). Solid tumors adapt to hypoxia by upregulating TF HIF-1α. Meanwhile, tumor-infiltrating natural killer (NK) cells are frequently dysfunctional in killing tumor cells (13). A recent single-cell RNA-seq study has shown that the depletion of HIF-1α in mouse NK cells elevates antitumor activity and inhibits tumor growth. Another study in a mouse model and clinical samples established that HIF-1α is a potent inhibitor of nuclear factor kappa B (NF-κB) signaling driven by interleukin (IL)-18 and the antitumor activity of tumor-infiltrating NK cells and, therefore, represents a potential immunotherapy target (14).

A few TFs are capable of motivating immunity cells to enhance their immune response, such as T-box expressed in T cells (T-bet) and forkhead box M1 (FoxM1). T-bet has been shown as one of the crucial TFs responsible for controlling the fate of both innate and adaptive immune cells, and its expression in many immune cells on mucosal surfaces can increase immunity (15). After treatment with Tet-derivative doxycycline to induce FOXM1, transgenic mice exhibited hepatic infiltration of macrophages (16). Despite the importance of TFs in regulating immune cell infiltration and immune response, a paucity of large-scale studies investigating the association of TFs with immune infiltration in pan-cancers remains.

In this study, we used the CIBERSORT technology on 100 paired bulk RNA-seq data from nine different patients with solid tumors to systematically explore the tumor-associated changes in immune cell composition. Moreover, we used weighted gene coexpression network analysis (WGCNA) to identify novel TFs strongly associated with tumor-associated immune cell infiltration. We found that TFs, including FOXM1, MYBL2, ERG, and TAL1, together with many immune-related genes, form several TF–immune-related gene expression networks (TF-iGENs). The analysis of The Cancer Genome Atlas (TCGA) data showed that the expression level of these genes involved in the same TF-iGEN were consistently associated with tumorigenesis and the survival of patients within multiple cancers. Public datasets from TCGA also validated the connection between TF-iGEN networks and immune infiltration. The expression quantitative trait locus (eQTL) analysis of exome-seq data showed that mutations were significantly more frequent in the regions flanking the TF genes than those with the non-TF genes. Our research presented a practical method for identifying TFs regulating immune infiltration, which could be potential new prognosis indicators and possible therapeutic targets in pan-cancers.

## Materials and Methods

### Tumor and Normal Dataset

We previously performed RNA-seq sequencing on 50 patients, including 100 RNA-seq data of the human tumor and tumor adjacent normal tissues covering nine cancer types, including six cervical squamous cell carcinoma (CSCC), six esophageal squamous cell carcinoma (ESCC), six gastric adenocarcinoma (GAC), six hepatocellular carcinoma (HCC), six lung adenocarcinoma (LUAD), six lung squamous cell carcinoma (LUSC), five papillary thyroid carcinoma (PTC), six small-cell lung carcinoma (SCLC), and three gastric signet-ring cell carcinoma (SRCC) (GEO accession: GSE87410) (**Table S1**).

### RNA-seq Data Processing and Analysis of Differentially Expressed Genes

For RNA-seq data, adaptors and low-quality bases were trimmed from raw sequencing reads using the FASTX Toolkit (Version 0.0.13). Reads shorter than 16 nt were discarded. Clean reads were aligned to the human GRCh38 genome by Tophat2 (17) allowing four mismatches. Uniquely mapped reads were ultimately used to calculate read number and reads per kilobase of exon per million fragments mapped (FPKM) for each gene. The software edgeR (18), which is specifically used to analyze differentially expressed genes (DEGs), was applied to screen the RNA-seq data for DEGs. The results were analyzed based on fold change (FC ≥2 or ≤0.5) and false discovery rate (FDR ≤0.05) to determine whether a gene was differentially expressed.

### Assessment of Tumor-Infiltrating Immune Cells

The CIBERSORT algorithm (8) (v1.03) was used with the default parameter for estimating immune cell fractions using FPKM values of each expressed gene. A total of 21 human immune cell phenotypes were analyzed in the study, including seven T cell types [CD8 T cells, naive CD4 T cells, memory CD4 resting T cells, memory CD4 activated T cells, T follicular helper cells, and regulatory T cells (Tregs)]; naive and memory B cells; plasma cells; resting and activated NK cells; monocytes; macrophages M0, M1, and M2; resting and activated dendritic cells; resting and activated mast cells; eosinophils; and neutrophils. An R package, Immunedeconv (19) (v2.0.2), that provided a unified interface to seven deconvolution methods was used for estimating immune cell fractions, while EPIC (20) was applied for estimating immune cell fractions.

### WGCNA Analysis

We applied WGCNA to fully understand the gene expression pattern in pan-cancers (21) to cluster genes having similar expression pattern with default parameters. All DEGs between the tumor and adjacent normal samples for each cancer type were used as input data. Eigengenes for each clustering module were used as the representative expression pattern of genes in each module. Module–trait associations were also investigated using WGCNA.

### TF-iGENs Analysis

Eigengene modules were selected to build a TF-iGEN. The expression levels of TF genes, LM22 marker genes, and immune-related genes (https://www.immport.org/shared/genelists) from the ImmPort database for each module were retained. Then, the Spearman correlation for each gene pair was calculated. Gene pairs with Spearman correlation coefficients >0.6 (or less than −0.6) and a corresponding *p* value (Benjamini–Hochberg corrected) <0.01 were considered to be significantly correlated. Then, TF–immune-related gene pairs or TF–LM22 gene pairs were retained when the TF–gene pair was found in the Encode TF target database (https://maayanlab.cloud/Harmonizome/dataset/ENCODE+Transcription+Factor+Targets) or TRRUST database (https://www.grnpedia.org/trrust/). The TF-iGEN was constructed using Cytoscape software.

### TCGA Dataset Analysis

Expression data for 33 cancer types from TCGA were analyzed using GEPIA2 (22), a web-based tool that compares gene expression between tumor and normal tissues from TCGA. Furthermore, GEPIA was used to comprehensively analyze the association of gene expression with overall survival (OS) in various types of cancer. GEPIA uses the Mantel–Cox test for hypothesis test. *p* < 0.05 was labeled as significant.

### Exome Sequencing

Exome sequencing of tumor and adjacent normal tissues was performed on 12 of the 50 patients having RNA-seq data (NCBI BIOPROJECT: PRJNA686225). Genomic DNA was extracted by the phenol chloroform extraction of nuclear pellets or using a Qiagen DNeasy Blood and Tissue kit (Qiagen). Purified genomic DNA was sheared to fragments of 100–500 base pairs, and 500 ng fragmented DNA was used for pair-end library preparation with a Truseq DNA library preparation kit (Illumina). After end-repair and 3′ dA overhanging, fragmented DNA was ligated to Truseq adaptors (Illumina) and amplified for 10 cycles. Liquid-phase sequence capture was performed using a NimbleGen SeqCap kit (Roche). The Truseq DNA libraries were denatured into single-stranded DNA and hybridized to SeqCap oligo pools. The bound DNA fragments were eluted and PCR amplified for another 10 cycles. Fragments corresponding to 200–400 bp were purified with AMpure Xp beads and stored at −80°C until use for sequencing. Enriched libraries were sequenced on an Illumina Nextseq 500 system (ABLife Inc.).

### Exome-seq Data Analysis

Adaptors were removed from raw reads using cutadapt (version 1.7.1) first, and then, reads were processed with the FASTX Toolkit (version 0.0.14) for trimming low-quality bases (qualities <20) and removing low-quality reads (<70% of read length with qualities <20). Then, N-containing reads were trimmed from N base. High-quality reads longer than 16 nt were aligned to the human genome (GRCh38) using BWA-MEM v 0.7.10-r789 (23) with default parameters. The resulting alignment was sorted by coordinates and further converted into binary alignment map (BAM) format using samtools v 1.6. The rmdup module of samtools was used to remove the duplicates from the data. The Genome Analysis Tool Kit (GATK) v3.5-0-g36282e4 (24) modules RealignerTargetCreator, Indel Re-aligner, and Base Re-calibrator were used to preprocess the alignments. During base quality recalibration, dbSNP variants and 1,000 genome variants were used as known sites. Target-capture efficiency metrics were determined using Picard HsMetrics. The realigned and recalibrated BAM file was used as an input to GATK HaplotypeCaller using the following parameters: genotyping_mode DISCOVERY -stand_emit_conf 10 -stand_call_conf 30. Finally, raw variant calls were soft filtered using GATK Variant Filtration based on the following parameters: Low Qual (30 < Q < 50). Variants were annotated using ANNOVAR (25).

### Detection of Somatic Mutations

Somatic mutations were identified using GATK Mutect2 in matched tumor and normal samples with default parameters. Candidate somatic mutations were further filtered based on gene annotations to identify those occurring in exon regions. The mutation landscape and visualization were created using the MafTools (v. 2.6.0) (26) in R software.

### eQTL Analysis

We performed additional eQTL analysis in 22 exome samples to validate the correlation between expression and mutation. We used Matrix eQTL (v2.3) (27) to test *cis*-eQTL associations, and parameters “useModel = modelLINEAR,” “errorCovariance = numeric(),” and “cisDist=1000000” were applied to assess the statistical significance between gene expression and single-nucleotide polymorphism (SNP) genotypes.

### Functional Enrichment Analysis

To sort out functional categories of DEGs, Gene Ontology (GO) terms and Kyoto Encyclopedia of Genes and Genomes (KEGG) pathways were identified using KOBAS v2.0 (28). Hypergeometric test and Benjamini–Hochberg false discovery rate (FDR) controlling procedure were used to define the enrichment of each term. To explore the biological processes and signal pathways related to immune system process pathway, enrichment analysis of genes in each WGCNA modules was performed by Gene Ontology and pathway analysis in Metascape (29).

### Statistical Analysis

All data presented were reproduced in at least three independent experiments. Statistical analysis was performed using the R software (https://www.r-project.org/) unless otherwise stated. Significance of differences was evaluated with either the Student’s *t*-test when only two groups were compared or the hypergeometric test for functional term enrichment analysis. No statistical methods were used to predetermine sample size (*p ≤ 0.05, **p ≤ 0.01, and ***p ≤ 0.001).

## Results

### Weighted Gene Coexpression Analysis Obtained Tumor-Enriched Modules Associated With the Estimated Proportion of Immune Cell Type Traits

**Figure 1:**
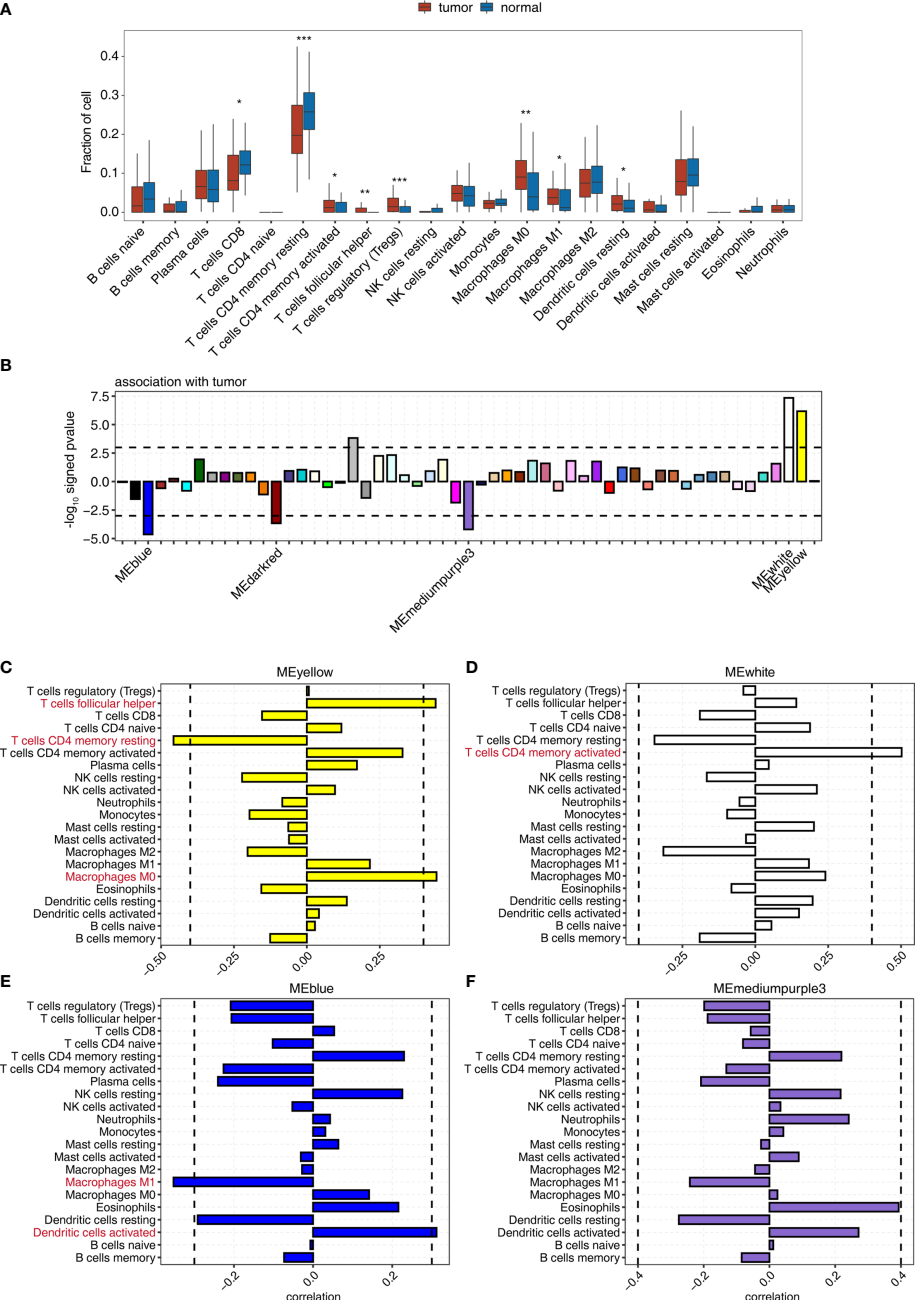
WGCNA determination of the correlation between the expression of module eigengenes whose expression was tumor deregulated and the change in the proportion of tumor-associated immune cells. **(A)** Boxplot showing the fraction of each immune cell type in tumor or normal samples; the significant difference in the immune cell fractions between these two groups was calculated using the Student’s *t*-test. *pvalue ≤ 0.05; **pvalue ≤ 0.01; ***pvalue ≤ 0.001 **(B)** Signed association of the module eigengene expression with tumor. Positive values indicate modules with increased expression in tumor samples. Negative values indicate modules with decreased expression in tumor samples. Dashed lines signify tumor-associated modules. **(C–F)** Correlation between immune cell population and the expression of module eigengenes from **(C)** MEyellow, **(D)** MEwhite, **(E)** MEblue, and **(F)** MEmediumpurple3. Dashed lines signify tumor-associated modules.

Tumor-infiltrating immune cells can shape the tumor microenvironment and are closely linked to immunotherapy. Using the CIBERSORT algorithm, we estimated the fraction of 21 subpopulations of immune cell types in 50 patients with CSCC, ESCC, GAC, HCC, LUAD, LUSC, PTC, SCLC, and SRCC (**Table S2**). Consistent with previous reports, the proportions of immune cells varied between different tumor types and their adjacent normal tissues (**Figure S1A**). Interestingly, the proportions of regulatory T cells (Tregs), M0 macrophages, T follicular helper cells, M1 macrophages, memory CD4 activated T cells, and dendritic cells in pan-cancer tissues were significantly (*p* < 0.05) higher than those in adjacent normal tissues, indicating their infiltration during tumorigenesis. On the contrary, the proportions of memory CD4 resting T cells and CD8 T cells were significantly (*p* < 0.05) lower (**Figure 1A**). The fluctuations in immune infiltration in tumor and adjacent normal samples across different cancer types were also exhibited using box plots (**Figure S1B**). We also applied EPIC, another tool, to estimate the proportions of different cell types from bulk gene expression data. We found that CD4+ T cells showed a lower proportion in tumors than in normal tissues, while cancer-associated fibroblasts showed a higher proportion in tumors than in normal tissues (**Figures S1C–E**).

Then, WGCNA was used to identify gene coexpression modules correlated with tumor and immune cell-type proportion. We identified 55 coexpressed gene modules using all DEGs between tumor and adjacent normal tissues of all nine cancer types (**Figure 1B**). The modules were named by different colors. Among them, 3/55, including MEblue, MEdarkred, and MEmediumpurple3 module, significantly negatively correlated (*p* < 0.001), and 2/55, including MEyellow and MEwhite module, significantly positively correlated (*p* < 0.001) with the tumor group (**Figures 1B**, **S2A**, and **Table S3**).

We examined the correlation between the expression of module eigengenes from MEyellow, MEwhite, MEblue, MEmediumpurple3, and MEdarkred, and the fraction of 21 immune cell populations to explore the association of immune cell composition dynamics with gene coexpression networks (**Figure S2B** and **Table S4**). The expression of these modules correlated with the infiltration of specific immune cell types (**Figures 1C–F**), indicating that we could use the gene expression from these modules to predict the fluctuation of the immune cell population in the tumor microenvironment. The significant correlation coefficients suggested the increased expression of the MEyellow module accompanied by the relatively high percentage of nonpolarized macrophages, M0 (**Figure 1C**); MEwhite module was most positively associated with memory CD4 activated T cells (**Figure 1D**). Similarly, improved infiltration of activated dendritic cells could be linked to the upregulated expression of MEblue module (**Figure 1E**). Nonetheless, the expression of ME mediumpurple3 and MEdarked modules showed a low association with infiltrating immune cells (**Figures 1F** and **S2C**). We also observed that MEsalmon4 eigengene expression was positively associated with the populations of Tregs, follicular helper T cells, and CD8 T cells (**Figure S2D**), and these eigengenes were strikingly overexpressed in HCC tumor tissues (**Figure S2E**). These results indicated that some gene coexpression networks strongly correlated with the proportion of specific immune cell types, contributing to the development of the specific immune cell population in the tumor microenvironment.

### Construction of TF-iGENs in MEyellow and MEblue Modules

TFs can control the expression of critical genes and thus play a prominent role in controlling the infiltration and functionality of immune cells (30, 31). We decided to identify the key TF regulators in MEyellow and MEblue modules that were more globally associated with tumor-infiltrating immune cell dynamics (see below). For tumor-associated genes in the MEyellow module, the KEGG analysis showed that their most enriched pathways were related to cell growth, including cell cycle, chromosome segregation, DNA replication, and DNA repair (**Figure S3A** and **Table S5**).

**Figure 2:**
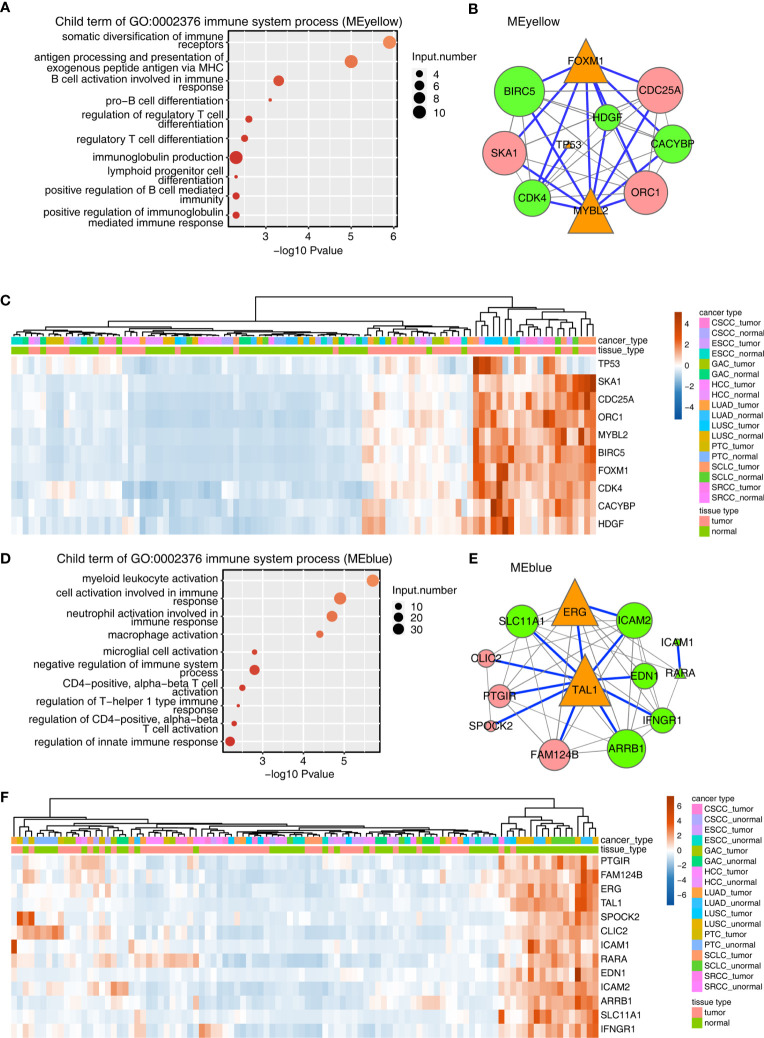
Construction of the TF–immune-related gene expression network in MEyellow and MEblue modules. **(A)** Metascape analysis was performed to cluster the tumor-associated genes in the MEyellow module. The parent GO term of the immune system process pathway was enriched, and its child GO terms were extracted and presented. **(A)** TF–immune-related gene expression network of the tumor-associated DEGs clustered in the MEyellow module. The expression levels of TF genes, LM22 marker genes, and immune-related genes were filtered. Then, we calculated the Spearman correlation for each gene pair. Gene pairs with Spearman correlation coefficients >0.6 (or less than −0.6) and a corresponding *p*value (Benjamini–Hochberg corrected) <0.01 were considered significantly correlated. Then, the TF–immune gene pair or TF–LM22 gene pair was filtered when the TF–gene pair was found in the Encode TF target database or the TRRUST database. The TF–immune-related gene expression network was built with Cytoscape in which the triangle represented TF, the green represented immune genes, and the red represented LM22 marker genes. Specifically, the connection of TF–immune-related genes with database support were illustrated with blue lines. Orange triangles indicated TF genes, green circles indicated immune-related genes, and pink circles indicated LM22 genes. Node size indicated the number of correlated genes in the gene network. **(A)** Expression profile of genes involved in TF–immune-related genes in **(B)**. **(D–F)** Same as 0 **(A–C)**, except that tumor-associated DEGs from the MEblue module were used.

Next, Metascape analysis was performed to cluster the tumor-associated genes in the MEyellow module; the parent GO term of the immune system process pathway was enriched, and its child GO terms were extracted and presented (**Figure 2A** and **Table S6**). These child GO pathways were mainly enriched in the differentiation and activation of immune cells, including T and B cells. These results were consistent with the positive correlation between MEyellow gene expression and follicular helper T cells and memory CD4 activated T cells, and with the negative correlation of MEyellow gene expression with CD4 memory resting T cells.

The aforementioned results encouraged us to analyze TF and the immune-related genes in the MEyellow module. To this end, a TF-iGEN was constructed based on both the correlation of gene expression between TFs and immune-related genes and also the binding potential of TFs on the correlated immune-related genes. Specifically, we first screened TFs, immune-related genes, and LM22 marker genes from the MEyellow module. Then, we calculated the Spearman correlation for each gene pair. Gene pairs with Spearman correlation coefficients >0.6 (or less than −0.6) and a corresponding *p* value (Benjamini–Hochberg corrected) <0.01 were considered significantly correlated. Then, TF–immune gene pair or TF–LM22 gene pair was filtered when the TF–gene pair was found in the Encode TF target database or TRRUST database (**Table S7**). The TF-iGEN was visualized in Cytoscape. Finally, we found that two hub TF genes, FOXM1 and MYBL2, were significantly associated with immune-related genes including *BIRC5*, *CACYBP*, *CDK4*, and *HDGF* (**Figure 2B**). TP53, another TF gene, was slightly associated with other immune-related genes (**Figure 2B**). We further demonstrated that the genes involved in TF-iGENs showed significantly higher expression levels in the tumor than in the adjacent normal tissues (**Figure 2C**).

A similar approach was then applied to analyze genes in the MEblue module; the most enriched KEGG pathways were different from the MEyellow module, mainly involving blood vessel development, extracellular structure organization, and cell adhesion (**Figure S3B** and **Table S8**). Child GO terms of the immune system process were mainly enriched in the activation of immune cells, such as myeloid leukocytes, neutrophils, macrophages, microglial cells, and T cells (**Figure 2D** and **Table S9**). We constructed TF-iGENs for the MEblue module (**Figure 2E**). Two hub TFs, TAL1 and ERG, were identified, which were significantly associated with immune-related genes, including *SLC11A1*, *EDN1*, *ICAM2*, *ICAM1*, *RARA*, *IFNGR1*, and *ARRB1*. Another TF gene, *RARA*, which was also involved in immune response pathways, was slightly associated with other immune-related genes. Different from that from the MEyellow module, the gene expression profile of TF-iGENs from the MEblue module showed a higher expression level in the adjacent normal samples than in the tumor (**Figure 2F**). These results indicated that the TF-iGENs derived from the two modules might carry different functionality.

### Validation of the TF-iGEN in TCGA Datasets

**Figure 3:**
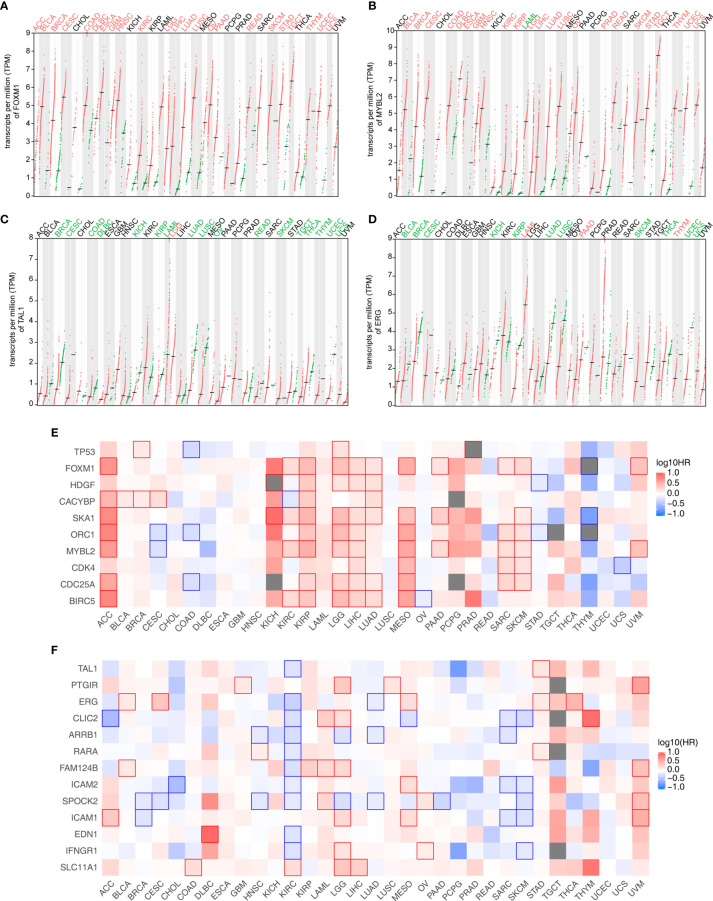
Analysis of the expression and its correlation with the survival rate of TF and immune-related genes identified in **Figure 2**. **(A–D)** Four TF expression profiles of 33 cancer datasets downloaded from the TCGA database. **(A)** FOXM1 and **(B)** MYBL2 were from the MEyellow module. **(C)** TAL1 and **(D)** ERG were from the MEblue module. Cancer names in red indicate that gene expression was significantly upregulated in tumors, and cancer names in green indicate that gene expression was significantly downregulated in tumors. **(E)** Association of the expression level of TF-iGENs in the MEyellow module with the patient survival rates in various types of cancer. **(F)** Association of the expression level of TF-iGENs in the MEblue module with the patient survival rates in various types of cancer.

We downloaded and profiled the expression of these candidate genes in 33 TCGA cancer datasets to further study the clinical relevance of the TF-iGEN in tumors (**Figures 3A–D**). FOXM1 and MYBL2 were significantly higher in tumors than in adjacent normal tissues in 70% (23/33) of cancers (**Figures 3A, B**). In contrast, TAL1 was significantly underexpressed in 52% (17/33) of tumors compared with normal tissues (**Figure 3C**), and ERG was significantly underexpressed in 33% (11/33) of tumors (**Figure 3D**). Another TF in the TF-iGEN from the MEyellow module, TP53, showed higher expression in pan-cancer compared with normal tissues (12/33) (**Figure S4A**). The differential expression of these six TFs was either never shown to be opposite or only shown to be opposite in one or a few cancer types (**Figures 3A–D** and **S4A, B**), suggesting a general role for these immune-related genes and TFs in many cancers. On the contrary, RARA was highly expressed in 6/33 cancers and lowly expressed in 5/33, demonstrating the controversial expression pattern (**Figure S4B**).

Since the expression of these networks closely correlated with the fluctuation in the immune cell population, we speculated that TF-iGENs had some prognostic value for patients with cancer. GEPIA2 analysis was further used to perform survival analyses for genes in the TF-iGENs. For each group, we conducted a heatmap to exhibit the survival analysis results across multiple cancer types (**Figures 3E, F**). In the heatmap, the red blocks denoted significantly high risk, and the blue ones denoted significantly low risk associated with higher gene expression. For the TF-iGENs from the MEyellow module, the high expression meant high risk. Especially in cancers such as ACC, KIRP, LGG, LIHC, LUAD, MESO, SARC, and SKCM, at least five genes in TF-iGENs showed consistent effects on the prognosis (**Figure 3E**). On the contrary, for the TF-iGENs from the MEblue module, high expression was more indicative of low risk (**Figure 3F**), suggesting that the survival time negatively correlated with the expression of these genes. These results demonstrated that some of the genes from TF-iGENs could serve as important indicators for the prognostic analysis of patients with cancers.

**Figure 4:**
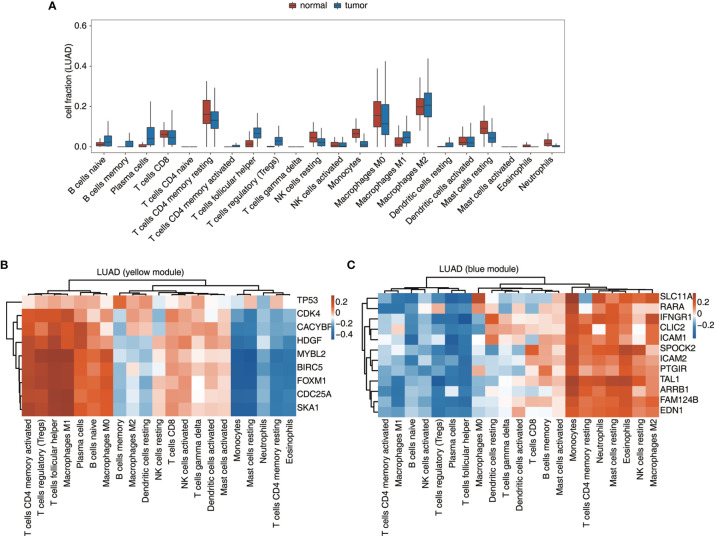
Validation of the TF-iGEN in TCGA datasets. **(A)** Boxplot showing the fraction of each immune cell type in tumor or normal samples using the LUAD dataset. **(B, C)** Correlation between immune cell population and the expression of genes from the TF–immune-related gene network in the **(B)** yellow module and the **(C)** blue module.

Besides expression analysis, two datasets from TCGA, LUAD, and LUSC were also used to validate the connection between TF-iGEN networks and immune infiltration. In our previous studies, compared with the elevated expression of genes from the MEyellow module, the fraction of some kinds of cells, such as CD4 memory activated T cells and M1 macrophages, increased in cancer tissues, while other cells such as CD4 memory resting T cells followed a different trend (**Figure S1B**). In LUAD and LUSC datasets, we observed a similar alteration of these immune cells from normal to cancer tissues (**Figures 4A** and **S5A**). Furthermore, Pearson’s correlation analysis was performed to test whether the expression of genes in TF-iGEN networks strongly correlated with the infiltration of immune cells. The expression levels of genes from the MEyellow module exhibited a positive correlation with CD4 memory activated T cells, Tregs, M1 macrophage M1, and so forth, and a negative correlation with monocytes, CD4 memory resting T cells, and so forth (**Figures 4B** and **S5B**). The expression of genes from the MEblue module also strongly correlated with specific immune cell fractions (**Figures 4C** and **S5C**). Together with our previous findings (**Figures 1C, E**), these results confirmed the potential usage of gene expression of TF-iGEN networks to predict immune infiltration. Furthermore, the expression pattern of all genes in two modules was validated using two datasets from TCGA (**Figures S6A, B**), and the results were similar to our findings.

### TF Genes in TF-iGENs Showed More Frequent *cis*-eQTL Compared With the Non-TF Genes

**Figure 5:**
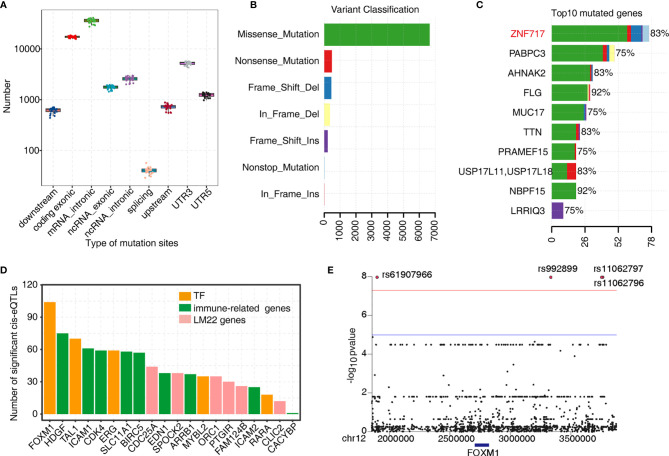
Analysis of the tumor somatic mutations in the TF–immune-related gene expression network. **(A)** Boxplot showing the relative abundance of SNVs found at different genomic locations. **(B)** Variant classification in the 24 samples. **(C)** Bar plot showing the top 10 mutant genes by the mutation number. **(D)** Barplot showing the number of significant cis-eQTLs of each gene from the TF–immune-related gene expression network of MEyellow and MEblue and was used to evaluate the mutation effects on TFs expression. **(E)** Manhattan plot of the cis-eQTL analysis of FOXM1.

As mentioned earlier, TF-iGENs strongly correlated with immune infiltration in the tumor microenvironment. Therefore, investigating the dynamic change in gene expression of TF-iGENs helped explain the altered immune cell population during tumorigenesis. TF-binding sites are frequently somatically mutated in cancer, leading to oncogenic activation (32, 33). To further explore whether the expression alteration of TF-iGENs was associated with somatic mutations, we performed exome sequencing on the paired samples from 24 of the 50 patients; RNA-seq data from all the 50 patients were available and analyzed in this study earlier (**Table S10**). Data were analyzed with R, and the results were visualized with “maftools” package. The distribution of somatic mutations in different genomic regions is shown (**Figure 5A**). We further classified these mutations according to different categories. As shown in **Figure 5B**, missense mutation was the most common type of variant classification. The bar plot showed the top 10 mutant genes by mutation number (**Figure 5C**), including *ZNF717* (83%), *PABPC3* (75%), *AHNAK2* (83%), *FLG* (92%), *MUC17* (75%), *TTN* (83%), *PRAMEF15* (75%), *USP17L11* (83%), *NBPF15* (92%), and *LRRIQ3* (75%). The waterfall chart was used to demonstrate the frequency mutation profile of the top 20 mutant genes (**Figure S6A**). The functional enrichment analysis on genes with mutations in more than 5/12 patients revealed that the genes were mainly involved in cell differentiation, proliferation, and apoptotic process (**Figures S6B, C**).

Next, we investigated the genetic basis of gene expression variation in TF-iGENs. Matrix eQTL was used for identifying eQTL based on the significance criterion. We classified eQTL with peaks within 1 MB of the transcript position and *p* < 0.01 as *cis*-eQTLs (**Table S11**). The number of significant *cis*-eQTLs of each gene from TF-iGENs is illustrated in **Figure 5D**. The number of *cis*-eQTLs helped evaluate the mutation effects on gene expression, including TF genes. Mutations were found to be more frequent in the regions flanking the TF genes than those with the non-TF genes (**Figure 5D**). A Manhattan plot of the *cis*-eQTL analysis of FOXM1 was further conducted (**Figure 5E**). Significant variants rs61907966, rs992899, rs11062797, and rs11062796 were identified. The enrichment of somatic mutations in tumor-deregulated TF genes than non-TF–immune-related genes supported the hypothesis that somatic mutation in key immune gene-related TFs might drive the tumor-specific immune cell infiltration.

## Discussion

In this study, we identified four key TFs significantly associated with tumor-infiltrating immune cells by analyzing 100 paired RNA-seq data obtained from 50 patients across nine cancer types. First, we estimated the fraction of 21 subpopulations of immune cells in each RNA-seq data using CIBERSORT and EPIC. Our results showed that the proportions of different types of T cells and macrophages were similarly altered in pan-cancer samples. The WGCNA analysis allowed us to further identify tumor-deregulated gene modules strongly associated with the tumor-induced change in the proportion of TIICs. On further constructing the TF-iGEN in two such modules, *FOXM1*, *MYBL2*, *TAL1*, and *ERG* were identified as key TFs regulating the expression of genes specifically involved in the immune cell differentiation or activation. Previous studies reported that *FOXM1*, *ERG*, and *MYBL2* played a role in tumor immune infiltration (16, 34, 35). The possible involvement of TAL1 in regulating tumor immune infiltration was first reported in the present study. We further verified the pan-cancer-regulated expression of these TF-iGENs in TCGA datasets derived from 33 cancer types. Additionally, the tumor-deregulated expression of these genes was strongly associated with the survival rate of pan-cancer patients. The eQTL analysis with exome-seq showed that mutations were significantly more frequent in the regions flanking the TF genes than those with the non-TF genes. These findings suggested that the TF-iGEN identified in this study might play some roles in regulating immune infiltration and affecting immune therapy and could serve as prognostic biomarkers of pan-cancers (30–32, 36, 37).

A previous study showed that CD4 memory activated T cells resembled the CD62 ligand low (CD62L-low) memory subset, showing rapid activation kinetics and high proliferative capacity (38). The tissue-resident dendritic cells that can capture antigen in peripheral tissues migrate into the lymph node and present peptides to the already activated CD4+ T cells (39), which motivates CD4 memory T cells to function in the microenvironment. In addition, many CD45RA-Foxp3 non-suppressive Treg cells in human cancer could differentiate into memory effector CD4+ T cells (40). This was consistent with our observation that the proportion of CD4 memory activated T cells in pan-cancer tissues was significantly higher than that in adjacent normal tissues, while the proportion of CD4 memory resting T cells was the opposite. Moreover, we demonstrated that both M0 and M1 macrophages were more enriched in tumor samples. Macrophages can switch from an unpolarized (M0) to a polarized (M1) phenotype in response to various stimuli such as tumors (41, 42), and M1 macrophages can exert proinflammatory functions. In contrast, M2 macrophages exert anti-inflammatory functions and facilitate tissue repair (43, 44). Therefore, it is reasonable that tumor induces M0 and M1, but not M2. In our study, M2 macrophages were higher in the non-cancer tissues.

Several TFs have been reported to regulate immune cell infiltration in cancer, such as FOXP1, FOXP3, and c-Maf (10, 12, 45). However, studies investigating TFs associated with immune cell infiltration in pan-cancers are lacking globally. Inkeles et al. previously integrated WGCNA gene modules with cell-type-specific gene signatures to investigate the genes and pathways associated with immune cell types that contributed to host defense and tissue injury at the site of infection in the different subtypes of leprosy (46). Inspired by this work, we used CIBERSORT and WGCNA to explore the correlation between the tumor-deregulated gene expression and tumor-infiltrating immune cell dynamics and further filtered out the TF-iGENs. Four key TFs (FOXM1, MYBL2, TAL1, and ERG) were identified as reliable key regulators in the network.

Previous studies revealed that forkhead box M1 (FoxM1) was overexpressed in HCC, and the induction of FOXM1 led to the hepatic infiltration of macrophages in mice (16). FOXM1 also weakened the promotion of T cell proliferation and depleted IL‐12 p70 in tumor‐bearing mice (47). Consistently, we found that FOXM1 was overexpressed in the tumor tissues of many different cancer types, and the expression dynamics of the eigengene module containing FOXM1 was positively associated with the increase in the proportion of follicular helper T cells and M0 macrophages. Moreover, the positive correlation between FOXM1 overexpression and the survival rate of multiple cancers was demonstrated. These findings suggested a more general role of FOXM1 in regulating immune infiltration, confirming the efficacy of our methods. The interactions between other TFs and immune infiltration were not studied much and comprehensively. MYBL2 was known to overexpress and associate with poor patient outcomes in many cancer entities (48); its expression positively correlated with CD4+ T cell infiltration but negatively correlated with B cell infiltration (35). In prostatic cancer, the expression of ERG was positively associated with CD204+ and CD3+ cell infiltration (34). The connection between TAL1 and the regulation of immune infiltration has not been reported due to insufficient data and hence deserves further investigation. Since the expression of these TFs strongly correlated with the infiltration of specific immune cell types, we believed that their important roles in regulating immune infiltration in the tumor microenvironment were revealed in our study.

The immune-related genes in the TF-iGEN are strongly associated with immune defense. Intercellular adhesion molecule 2 increases the antitumor immunity by accelerating the infiltration of immature myeloid dendritic cells in the tumor epithelium, followed by cellular immune responses, and promoting the susceptibility of the tumor cells to cytotoxic T-cell-mediated cytolysis in intraductal papillary mucinous adenoma (49). The solute carrier family 11a member 1 modulates macrophage activation by regulating immune-inflammation genes in macrophages (50); these include tumor necrosis factor-α (TNF-α), interferon gamma (IFN-γ), and IL-1 (51), and major histocompatibility complex (MHC) class II expression (52). The inhibitor of cyclin-dependent kinases 4 (CDK4), which has already been approved by the Food and Drug Administration (FDA) for the treatment of breast cancer (53, 54), markedly repressed the proliferation of Tregs (55). This finding was consistent with our research that the expression of CDK4 was positively associated with the population of Tregs. Therefore, the pan-cancer-associated TF-iGEN is functionally related to immune infiltration, which likely represents a mechanism underlying the altered immune cell contexture in the tumor microenvironment. Moreover, the expressions of many TFs and immune-related genes in the networks increase or decrease significantly during cancerization, suggesting that individually or combined, they can serve as indicators to predict the prognosis situation. It was remarkable that most of the genes in our TF-iGENs showed a significant prognostic value in brain lower-grade glioma, adrenocortical carcinoma, liver hepatocellular carcinoma, sarcoma, and kidney renal clear cell carcinoma, which demonstrated the clinical relevance of our results.

Mutations in both coding or non-coding areas (56) could affect transcriptional regulatory mechanisms. Many TFs have been found to have a high mutation frequency and are related to the occurrence and development of tumors. ATBF1 encodes a transcription factor that could inhibit cell proliferation. ATBF1 messenger RNA (mRNA) is abundant in normal prostates but more scarce in approximately half of prostate cancers tested. Frequent somatic mutations of the transcription factor ATBF1 in human prostate cancer were found, many of which impair ATBF1 function (57). RUNX1, another transcription factor mutated in breast cancer, was found as a key regulator of the ER+ luminal lineage whose disruption may contribute to the development of ER+ luminal breast cancer when under the background of either TP53 or RB1 loss (58). Mutations of TP53 (59) significantly correlated with the programmed death 1, programmed death-ligand 1, and programmed death-ligand 2 axis (60–62), which had a significant influence on immune infiltration. The eQTL analysis in the genes of our TF-iGEN showed that mutations were significantly more frequent in the regions flanking the TF genes than those with the non-TF genes, supporting a potential mechanism that somatic mutation in key immune gene-related TFs might drive the altered expression of immune infiltration during cancerization and consequently altered immune infiltration. This hypothesis requires further investigation.

## Data Availability Statement

The data reported in this paper have been deposited with the NCBI Gene Expression Omnibus (GEO, https://www.ncbi.nlm.nih.gov/geo/) under accession number GSE87410.

## Ethics Statement

The studies involving human participants were reviewed and approved by the ethics committee of the China–Japan Union Hospital of Jilin University. The patients/participants provided their written informed consent to participate in this study.

## Author Contributions

LS, XC, and YZ conceived and supervised the study. LL, JY, and HS performed the experiments. CC, QW, and QZ performed data analyses. LL, QZ, and CC wrote the manuscript. All authors contributed to the article and approved the submitted version.

## Funding

This work was funded by the Research Foundation of Jilin Provincial Science & Technology Committee (Nos. 20200201135JC and 20200404124YY) and the Scientific Research Foundation of Jilin Province (20200601010JC and 20190701061GH).

## Conflict of Interest

QZ, CC, QW, WQ, YX, and YZ were employed by the company ABLife BioBigData Institute.

The remaining authors declare that the research was conducted in the absence of any commercial or financial relationships that could be construed as a potential conflict of interest.

## Publisher’s Note

All claims expressed in this article are solely those of the authors and do not necessarily represent those of their affiliated organizations, or those of the publisher, the editors and the reviewers. Any product that may be evaluated in this article, or claim that may be made by its manufacturer, is not guaranteed or endorsed by the publisher.
