# Supplementary material for: Analysis of Bulk RNA Sequencing Data Reveals Novel Transcription Factors Associated With Immune Infiltration Among Multiple Cancers
Source: Front Immunol. 2021 Aug 20;12:644350. doi: 10.3389/fimmu.2021.644350 (PMC8417605; doi:10.3389/fimmu.2021.644350)
Supplement: Supplementary file 1 [file DataSheet_1.pdf]

Supplementary Materials

Analysis of bulk RNA sequencing data reveals novel transcription factors associated with immune infiltration among multiple cancers

Lei Liu[1†], Chao Cheng[4†], Qiuchen Zhao[4,5†], Jingwen Yi[1], Hongyan Sun[2], Weili Quan[4], Yaqiang Xue[4], Luguo Sun[1,3*], Xianling Cong[2*], Yi Zhang[4*]

[1]National Engineering Laboratory for Druggable Gene and Protein Screening, Northeast Normal University, Changchun 130024, China

[2]Tissue Bank, China-Japan Union Hospital, Jilin University, Changchun 130033, China

[3]Research Center of Agriculture and Medicine gene Engineering of Ministry of Education, Northeast Normal University, Changchun 130024, China

[4]ABLife BioBigData Institute, Wuhan, Hubei 430075, China

[5]College of Life Sciences, Wuhan University, NO.299 Ba Yi Avenue, Wuchang, Wuhan 430072, China

†These authors contributed equally to this work.

*Corresponding authors: sunlg388@nenu.edu.cn; congxl888@hotmail.com; yizhang@ablife.cc

TABLE OF CONTENTS

SUPPLEMENTARY FIGURES..3

SUPPLEMENTARY TABLES 11

SUPPLEMENTARY FIGURES

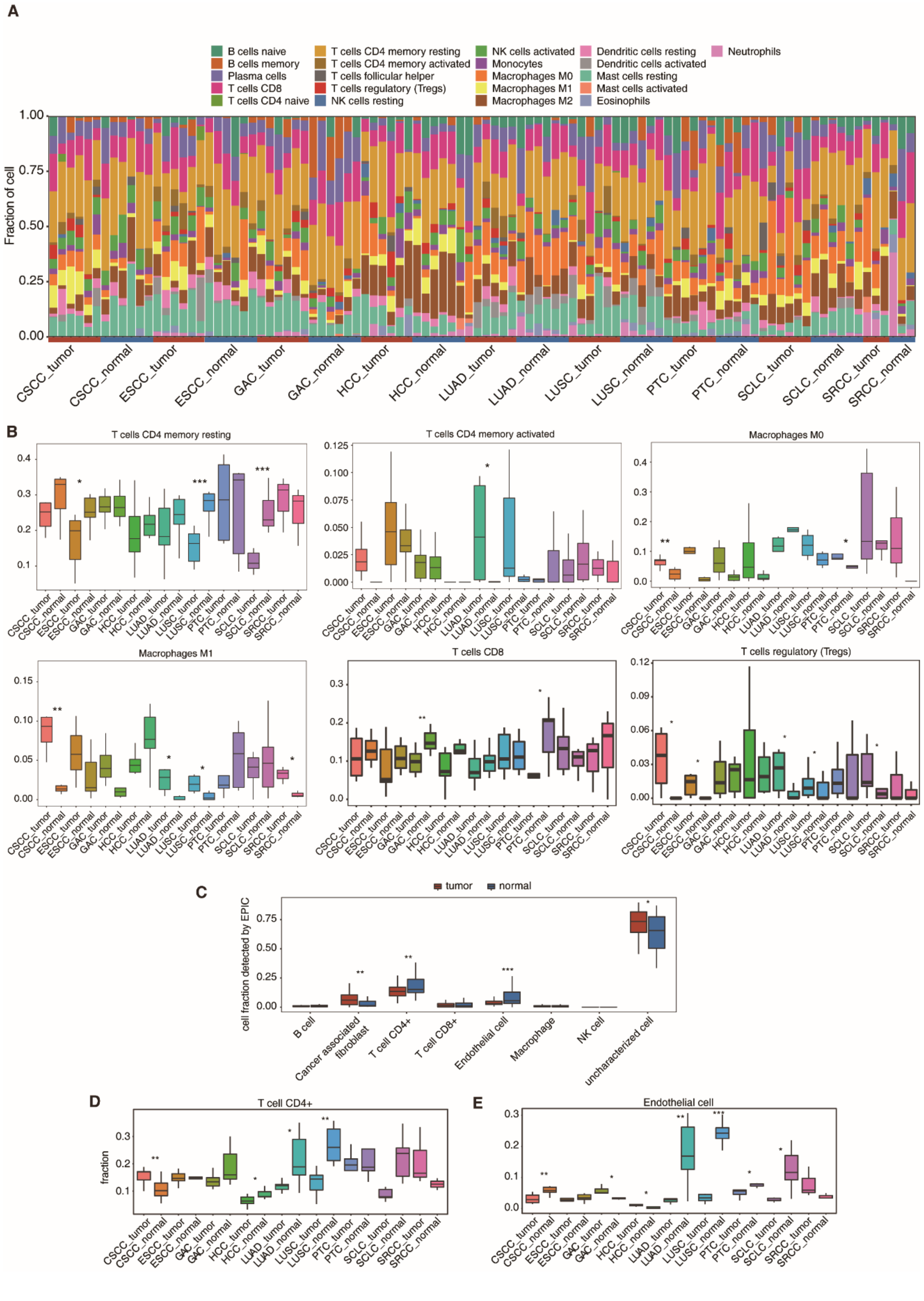

Figure S1. Analysis of the dynamics of cell population of tumor and adjacent normal tissues from multi-cancer types.

A. Fractions of different immune cells estimated by Cibersort in each sample. The 100 RNA-seq transcriptomes from 50 pairs of the tumor and adjacent normal tissues covering 9 different cancer types were analyzed.

B. Box plots showing proportion of four cell type in tumor or normal samples from different cancer type.

C. Boxplot showing the fraction of each immune cell type in tumor or normal samples using EPIC, the significant difference in the immune cell fractions between these two groups was calculated using Student' t-test.

D-E. Box plots showing proportion of two cell type in tumor or normal samples using EPIC from different cancer type.

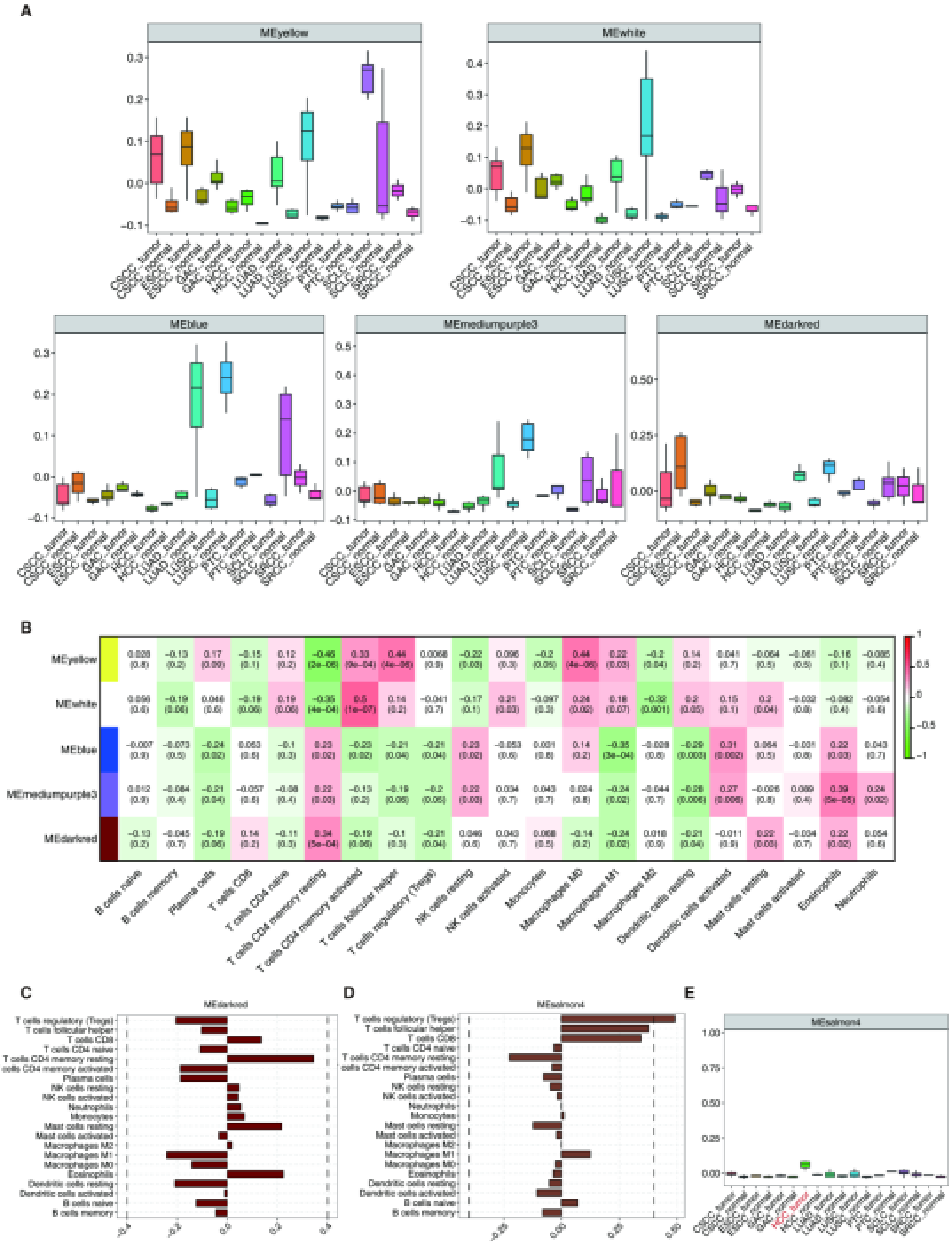

Figure S2. WGCNA was used to determine the correlation of module eigengenes of DE genes to tumor state and cell type proportion.

A. Boxplot showing expression of genes in tumor and normal samples of each cancer type from the five tumor-associated modules.

B. Module-trait associations as computed by an LME model with all factors on the x axis used as covariates. All Pearson’s correlation value and p values are displayed.

C-D. Correlation of module eigengenes from MEdarkred (C) and MEsalmon4 (D) with cell population. Dashed lines signify tumor-associated modules.
E. Boxplot showing expression of genes in tumor and normal samples of each cancer type from the MEsalmon4 module which is significantly associated with HCC cancer.

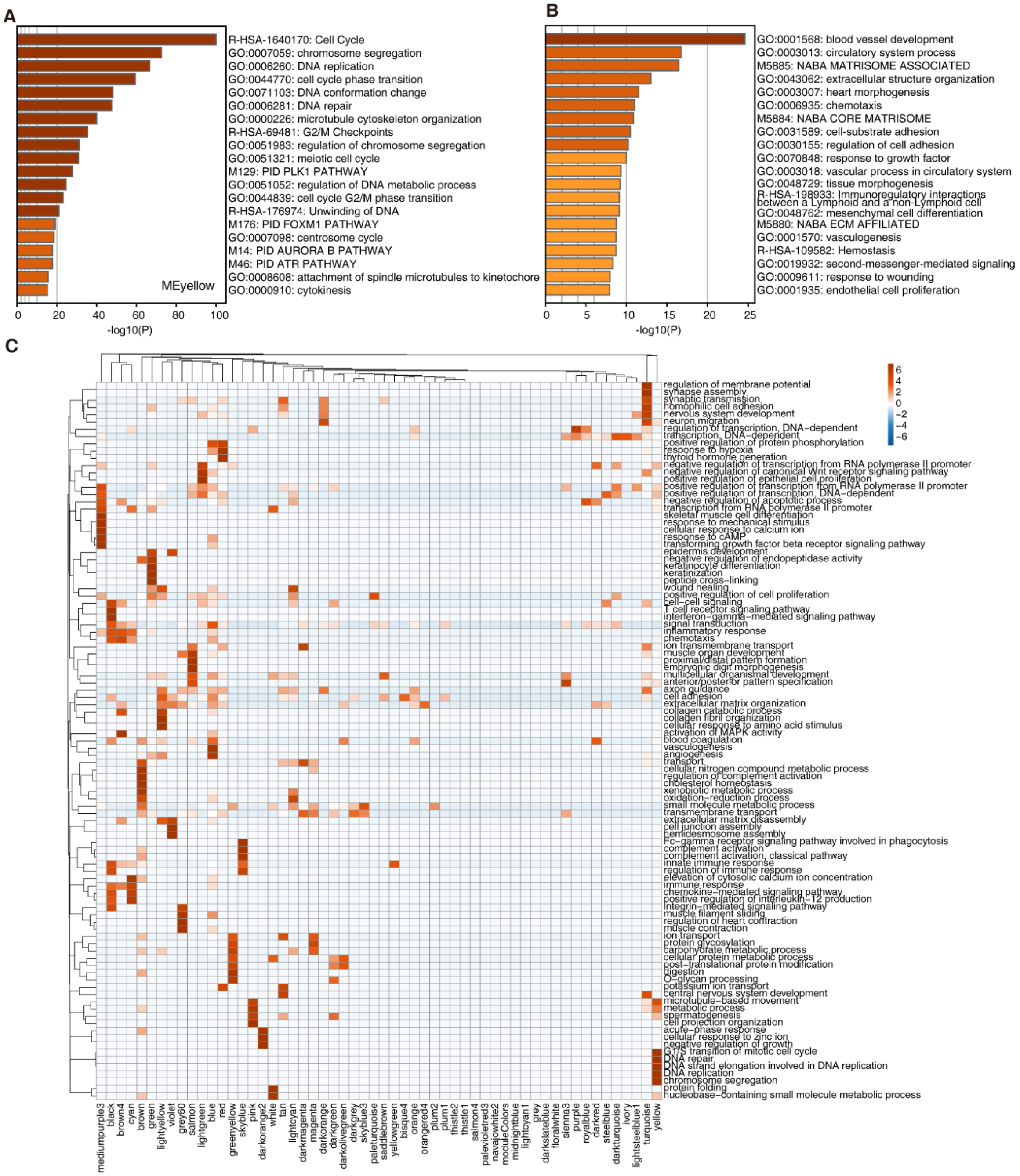

Figure S3. Construction of TF-immune gene network in MEyellow and MEblue modules.

A. Bar graph of enriched terms across gene lists of MEyellow module, colored by pvalue.
B. Bar graph of enriched terms across gene lists of MEblue module, colored by pvalue.
C. GO terms heatmap for each module constructed by running WGCNA. -log10pvalue of top5 terms of each module were combined and used for drawing heatmap.

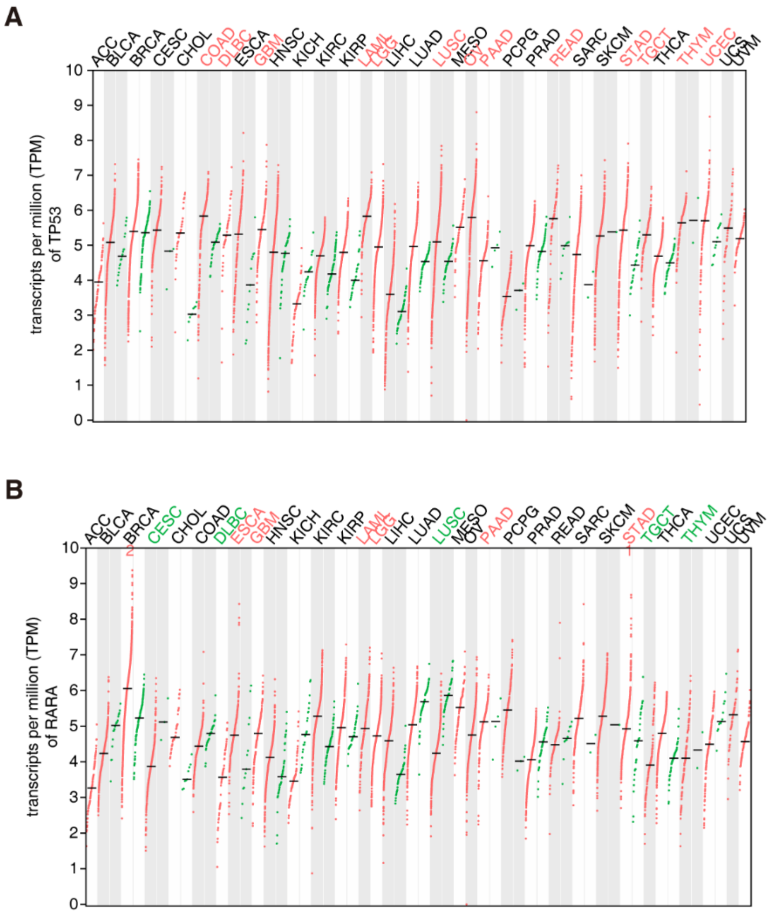

Figure S4. The expression of TF genes from the networks shown in Figure2.

The expression profile of TFs TP53 (A) and RARA (B) in 33 cancers. The data were obtained from TCGA database.

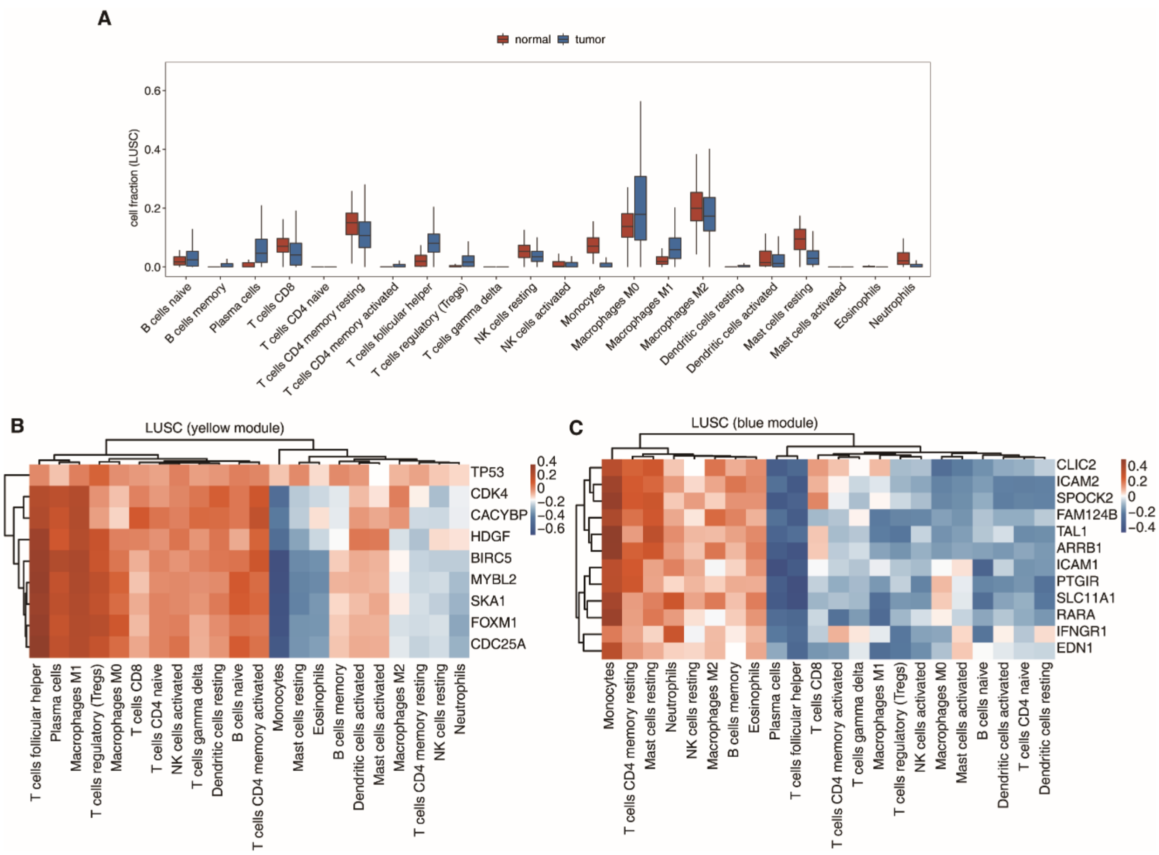

Figure S5. Validation of TF-iGEN network in TCGA datasets.

A. Boxplot showing the fraction of each immune cell type in tumor or normal samples using LUSC dataset.

B-C. Correlation between immune cell population and the expression of genes from TF-immune gene network in yellow module (B) and blue module (C).

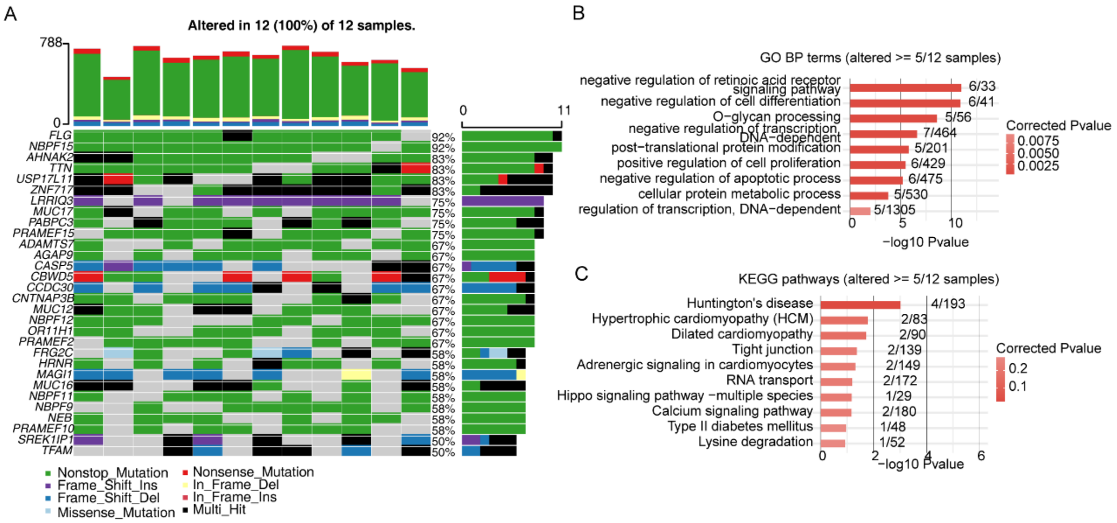

Figure S6. Overview of variants.

A. Waterfall plot showing top 30 alteration landscape for 12 tumor and normal tissues.

A. Top 10 most enriched GO terms (biological process) of genes with mutations altered in more than 5 in 12 samples.

B. Top 10 most enriched KEGG pathways of of genes with mutations altered in more than 5 in 12 samples.

SUPPLEMENTARY TABLES

Supplemental Table 1. Information of the 100 RNA-seq data from 50 patients, including 9 cancer types.

Supplemental Table 2. Fraction of in 21 subpopulations of immune cell types in 50 patients.

Supplemental Table 3. Correlation of WGCNA modules with tumor group.

Supplemental Table 4. Correlation of WGCNA modules with immune cell type population.

Supplemental Table 5. Top15 enrichment pathways by Metascape with genes in MEyellow module.

Supplemental Table 6. Enriched child GO terms of the immune System process pathway by Metascape with genes in MEyellow module.

Supplemental Table 7. Gene pair list and correlation in TF-iGENs.

Supplemental Table 8. Top15 enrichment pathways by Metascape with genes in MEblue module.

Supplemental Table 9. Enriched child GO terms of the immune System process pathway by Metascape with genes in MEblue module.

Supplemental Table 10. Information of the 22 Exome-seq data.

Supplemental Table 11. cis-eQTLS of genes in TF-iGENs.
